# Multi-Functional Potential of Presumptive Lactic Acid Bacteria Isolated from Chihuahua Cheese

**DOI:** 10.3390/foods9030276

**Published:** 2020-03-03

**Authors:** María Georgina Venegas-Ortega, Adriana Carolina Flores-Gallegos, Cristóbal Noé Aguilar, Raúl Rodríguez-Herrera, José Luis Martínez-Hernández, Guadalupe Virginia Nevárez-Moorillón

**Affiliations:** 1 Research Group of Bioprocesses and Bioproducts. Department of Food Research, School of Chemistry, Universidad Autónoma de Coahuila, 25280 Saltillo, Coahuila, Mexico; 2 Facultad de Ciencias Químicas, Universidad Autónoma de Chihuahua, Circuito Universitario S/N, Campus Universitario II, 31125 Chihuahua, Mexico

**Keywords:** food pathogens, proteolytic, lipolytic, phenotypic characterization, lactic acid bacteria

## Abstract

The multifunctional properties of autochthonous lactic acid bacteria can be of use for enhancing the sensorial properties of food, as well as in food preservation. An initial screening for antimicrobial, proteolytic, and lipolytic capacities was done in 214 presumptive lactic acid bacteria isolates obtained from Chihuahua cheese manufacturing and during a ripening period of nine months. The antimicrobial screening was done by spot-on-the-lawn tests, using *Listeria monocytogenes* and *Escherichia coli* as indicator microorganisms; proteolysis was tested in casein-peptone agar and lipolysis in Mann–Rogosa–Sharpe (MRS)-tributyrin agar. More than 90% of the isolates hydrolyzed the casein, but only 30% hydrolyzed tributyrin; the inhibition of *L. monocytogenes* in the spot-on-the-lawn assay was used to select 39 isolates that had a bigger inhibition zone (>11.15 mm ± 0.3) than the control (Nisin producer *Lactococcus lactis* BS-10 Chr Hansen). The selected isolates were grown in MRS to obtain the neutralized cell-free supernatants and verify their antimicrobial activity by agar diffusion and the percentage of growth inhibition techniques. The selected isolates were also growth in casein peptone broth, and the cell-free supernatants were used for the determination of antioxidant activity by the radical scavenging of 1,1-diphenyl-2-picrylhydrazyl (DPPH) and 2,2-azinobis (3-ethylbenzothiazoline-6-sulfonate) (ABTS) techniques. The results were analyzed to identify similarities by cluster analysis, based on their antimicrobial and antioxidant capacities. The isolates were arranged into six clusters; one cluster that included 12 isolates demonstrated *L. monocytogenes* (784–2811 mm^2^/mL AU by agar diffusion assay) and *E. coli* (41%–47% growth inhibition) antimicrobial activity. The isolates clustered in these groups also showed competitive inhibition of both radicals (11%–19% of DPPH and 50%–60% of ABTS). The isolates from cluster one were also identified by 16S rDNA amplification and were identified as *Enterococcus faecium*. Traditional products such as Chihuahua cheese can be a source or lactic acid bacteria with metabolic properties that can be used in food preparation and preservation.

## 1. Introduction

Lactic acid bacteria (LAB) are microorganisms widely dispersed in many environments that have shown desirable characteristics for the food, pharmacy, cosmetics, veterinary, and agricultural industries. LAB have been used from ancient times owing to their fermentative properties, but they are also extensively studied lately for the multi-functional capacities, which include enzymatic and metabolite production, which are of importance in the food industry. For instance, LAB-associated proteolysis and lipolysis are related to flavor and texture attributes in dairy products [1]. LAB with different enzymatic activities can have multiple applications; in the case of proteolytic LAB strains, the generation of bioactive peptides from different protein matrices has gained considerable attention [2]. The bioactivities of these peptides include antioxidant and antimicrobial activity, immunomodulation, angiotensin-converting-enzyme inhibition, as well as anti-thrombotic capacity [3]. In vitro studies have shown that antioxidant peptides from LAB fermentation can participate as radical scavengers, lipid peroxidation inhibitors, metal chelators, and maintain cell viability in oxidative stress conditions [4,5,6]. Thus, bioactive peptides with antioxidant properties can be multi-functional as they can be used in food preservation and can also serve as therapeutic/preventive agents against cancer. On the other hand, lipase and esterase could be microbially produced, including LAB, which are commercially significant not only in the food industry, but also in the cosmetic and pharmaceutic industries. Most of the LAB functional properties including enzymatic activities have been studied mainly in *Lactobacillus* or *Lactococcus*; however, less is known about proteolytic system of *Enterococcus* strains, which also show higher lipolytic activities than other LAB genera [7].

LAB can also produce antimicrobial metabolites used for their environmental survival. These compounds include organic acids, hydrogen peroxide, diacetyl, biosurfactants, and bacteriocins, the latter being peptidic compounds produced in an auto-regulated genetic expression system that includes the structural, modifiers, translocation, regulation, and self-immunity genes (to give protection to the producer strain) [8]. Bacteriocins spectrum includes bacteria closely related to the producer strain and, in some particular cases, other types of bacteria and even fungi [9]. Despite the existence of many antimicrobial compounds in the market, bacteriocins participate as natural compounds that may be potentially accepted in GRAS (generally recognized as safe) status when they are produced by LAB. The bacteriocins nisin and pediocin are already in this status [10].

Few investigations have proposed the use of LAB as multi-functional, owing to their metabolic capacities. Ramakrishnan et al. [11] exploited the metabolic activities of *Enterococcus faecium* and *Enterococcus durans* by demonstrating the protease, lipase, and bacteriocin production from fish wastes. Sharma and Saharan [12] simultaneously produced bacteriocin and biosurfactants from *Lactobacillus casei*, to provide a potentially synergistic food preservation product. The challenge is to find the matrix that not only supports the production of bacteriocins, but also contains proteins that can serve as precursors of bioactive peptides. By producing extracellular antioxidant and antimicrobial peptides during LAB growth, a potential additive can be obtained to be used in the food processing, pharmaceutical, veterinary, and cosmetics industries. Therefore, this work was aimed at screening presumptive LAB isolates from the Chihuahua cheese manufacturing process to evaluate their enzymatic, antimicrobial, and antioxidant properties.

## 2. Material and Methods 

### 2.1. Bacterial Isolates and Culture Conditions

A total of 214 presumptive LAB isolates were recovered from a bacterial collection conserved at −20 °C, which contained previously isolated bacterial cultures from three Chihuahua cheese factories; samples were taken during three seasons within a year, from different parts of the manufacturing process as well as during the ripening period. Isolates from the bacterial collection were obtained from plates of Mann–Rogosa–Sharpe (MRS), M17, and Elliker Agar (BIOXON, Mexico City, Mexico), incubated under a reduced oxygen atmosphere (BD Gas Pack CO_2_, Franklin Lakes, NJ, USA), as well as Kanamycin Esculin Azide Agar and Bile Esculin Agar (DifcoTM, Franklin Lakes, NJ, USA), from enumeration plates of samples taken at different Chihuahua cheese manufacturing steps [13,14]. Isolates were selected based on their colonial and microscopic morphology and catalase reaction. *Lactococcus lactis* BS-10 Chr. Hansen was used as a control strain because of its capacity for Nisin production. *Listeria monocytogenes* ATCC 19112 and *Escherichia coli* ATCC 25922 were used as indicator strains for the antimicrobial assays.

Broth and agar medium of MRS and Trypticase Soy Agar 1.5% yeast extract (TSAYE) (BIOXON, Mexico City, Mexico) were used for recovery of presumptive LAB and indicator strains, respectively, as well as for the antimicrobial screening and confirmation assays. Temperatures for recovery incubation was 26 °C for mesophilic LAB and 36 °C for thermophilic LAB, *E. coli,* and *L. monocytogenes*. Once recovered, all presumptive LAB isolates were incubated in 30 °C.

### 2.2. Screening for Antimicrobial Activity by Spot-On-The-Lawn (SOTL) Method

The SOTL method was done according to Lewus and Montville [15] with some modifications. A 10 μL aliquot from an overnight culture of the presumptive LAB was spotted in MRS and TSAYE agar plates. After 24 h of incubation (30 °C), the plates were overlaid with TSAYE agar seeded with 10^8^ cells of each indicator strain in 10:1 relation liquid agar/inoculum. The TSAYE was maintained at 40 °C in a water bath until the inoculation. The overlaid plates were incubated at indicator strains conditions (37 °C, 24 h), and the inhibition (clear zone) was measured afterwards, using a digital Vernier caliper. The presumptive LAB isolates that presented a clear zone larger than the control strain (11.15 ± 0.3 mm) were selected for further studies.

### 2.3. Neutralized Cell-Free Supernatants (NCFSs)

Twenty-five milliliters of MRS broth in Erlenmeyer Flasks (125 mL) was inoculated (0.1% *v*/*v*) with an overnight culture of the selected presumptive LAB isolates. Flasks were incubated (30 °C, 18 h, 140 rpm) and then transferred to conical tubes to be centrifuged (10,000 *×g*, 20 min, 4 °C) (Centrifuge Eppendorf 5804R, Eppendorf, Hamburg, Germany). Supernatants were obtained by recovering the decanted medium, and then it was neutralized (NaOH 3M) until pH = 6.5 was reached. The neutralized supernatants were filtered (0.22 μm) to obtain the NCFSs for antagonistic studies.

### 2.4. Antimicrobial Activity By Agar Diffusion Assay (ADA)

Antimicrobial activity of NCFSs was determined by ADA, according to Avaiyarasi et al. [16] with some modifications. TSAYE agar was seeded with 10^8^ cells of each indicator strain in 10:1 relation liquid agar/inoculum. The TSAYE was maintained at 40 °C in a water bath until inoculation. Once the agar was solidified, 8 mm holes were punched with a sterilized cork boarer; 50 μL of each NCFSs was added to each well. Plates were incubated (36 °C, 24 h) and clear zones around the wells were measured to calculate the antimicrobial activity, which was expressed as arbitrary units (AU) with the following equation.

(1) $$\mathrm{AU}=\frac{\mathrm{Inhibition}\;\mathrm{area}\;\left({\mathrm{mm}}^{2}\right)-\mathrm{Well}\;\mathrm{area}\;\left({\mathrm{mm}}^{2}\right)}{\mathrm{Volume}\;\mathrm{sample}\;\left(\mathrm{ml}\right)}={\mathrm{mm}}^{2}/\mathrm{ml}$$

### 2.5. Antimicrobial Activity By Microplate (MP) Assay

The antimicrobial activity of NCFSs was determined by MP. Microplates of 96 wells were loaded using the following strategy.

(a) Culture with treatment (CWT): 100 μL of TSAYE broth, 50 μL of NCFSs, and 30 μL of each indicator strain (10^8^ cells/mL).
(b) Culture without treatment (CWOT): 100 μL of TSAYE broth, 50 μL of MRS broth, and 30 μL of each indicator strain (10^8^ cells/mL).
(c) Blank: 100 μL of TSAYE broth, 50 μL of MRS broth, and 30 μL of saline water (0.9% *w*/*v*).

Absorbance (Abs) was measured (595 nm) at 0 h and 24 h after incubation (36 °C). Antimicrobial activity was calculated and expressed as a percentage of inhibition (% Inh) using the following equation.

(2) $$\%\;\mathrm{Inhibition}=100-\left(\frac{\mathrm{Abs}\;\mathrm{CWT}\;24h-\mathrm{Abs}\;\mathrm{CWT}\;0h}{\mathrm{Abs}\;\mathrm{CWOT}\;24h-\mathrm{Abs}\;\mathrm{CWOT}\;\mathrm{Oh}\;}*100\right)$$

### 2.6. Qualitative Determination of Proteolytic and Lipolytic Activity

From an overnight culture of presumptive LAB isolates, 3 μL was spotted in Petri plates containing 5 g/L of casein peptone agar (Bioxon, Becton Dickinson, Cuautitlan-Izcalli, Mexico) supplemented with 1.5% skimmed milk (Nestle, Mexico City, Mexico). Proteolysis was detected after incubation (30 °C, 24 h) by a clear zone around the colony. For lipolysis determination, a 3 μL aliquot from an overnight presumptive LAB culture was spotted in Petri plates containing MRS agar supplemented with 1 mL/L of tributyrin (Sigma-Aldrich, St. Louis, MO, USA). Lipolysis was detected after incubation (30 °C, 48 h) by a clear zone around the colony [17].

### 2.7. Cell-Free Supernatant Hydrolyzes (CFSH)

Twenty-five milliliters of casein peptone broth (15 g/L) supplemented with 1.5% (sterilized) skimmed milk was placed in Erlenmeyer flasks (125 mL) and inoculated (0.1% *v*/*v*) with an overnight culture of the selected presumptive LAB isolates. Flasks were incubated (30 °C, 18 h, 140 rpm) and then transferred to conical tubes to be centrifuged (10000 *×g*, 20 min, 4 °C (Centrifuge Eppendorf 5804R). Supernatants were freeze-dried (Free Zone Triad Freeze Dryer, Labconco, Kansas City, MO, USA) before further analysis.

### 2.8. Scavenging of 1,1-Diphenyl-2-Picrylhydrazyl (DPPH) Free Radical 

For the DPPH radical scavenging activity, the method used is based on Pownal et al. [18] and Nicklisch and Waite [19] with some modifications. Freeze-dried samples were dissolved in 0.1 M phosphate buffer (pH 7.0) to a concentration of 1 mg/mL, and 100 μL of each solution was added to 100 μL of 100 μM DPPH solution. The solution was prepared in 99% methanol at a concentration of 2 mM and then dissolved (1:20) in a solution of 0.1 M citrate buffer supplemented with 0.3% Triton X-100 (Probiotek, Ontario, Canada). The mixture was kept for 40 min at room temperature, and then the absorbance was measured at 517 nm (Epoch Microplate Spectrophotometer, Bio Tek, Winooski, VT, USA). A lower absorbance corresponds to a higher DPPH radical-scavenging activity (% DPPH). The scavenging effect was expressed, as shown in the following equation.

(3) $$\%\;\mathrm{DPPH}=\left(\frac{\mathrm{Blank}\;\mathrm{absorbance}-\mathrm{Sample}\;\mathrm{absorbance}}{\mathrm{Blank}\;\mathrm{absorbance}}\right)*100$$

Trolox (6-hydroxy- 2,5,7,8 tetramethyl chroman-2-carbocyclic acid) (Sigma-Aldrich, Saint Louis, MO USA) was used as control.

### 2.9. Scavenging of 2,2-Azinobis (3-Ethylbenzothiazoline-6-Sulfonate) Radical Anion (ABTS-) 

The assay was conducted according to the method of Re et al. [20] with slight modifications. ABTS+ was generated by mixing ABTS stock solution (7 mM) and potassium persulfate (2.45 mM) in distilled water, and the mixture was kept at room temperature in the dark for 16–17 h. The assay was done in a 96-well plate and the sample was used at 1 mg/mL. The reaction was initiated by mixing 10 μL of the sample solution or diluted Trolox with 190 μL of diluted ABTS+ (absorbance 0.7 at 734 nm) (Epoch Microplate Spectrophotometer, Bio Tek). The decrease in absorbance was measured at 734 nm after 40 min of incubation. The scavenging effect was expressed as percentage of radical scavenging activity.

(4) $$\%\;\mathrm{ABTS}=\left(\frac{\mathrm{Blank}\;\mathrm{absorbance}-\mathrm{Sample}\;\mathrm{absorbance}}{\mathrm{Blank}\;\mathrm{absorbance}}\right)*100$$

Trolox (6-hydroxy- 2,5,7,8 tetramethyl chroman-2-carbocyclic acid) (Sigma-Aldrich, Saint Louis, MO USA) was used as control.

### 2.10. Genomic DNA Extractions from Presumptive LAB Cultures

From overnight cultures of selected presumptive LAB isolates, genomic DNA was obtained by the enzymatic lysis assay [21,22]. DNA was extracted from a 2 mL bacterial cultured in MRS (30 °C, 24 h); cells were centrifuged (10 min, 10,000 *×g*); 100 μL of lysozyme and 10 μL of proteinase K were added to the pellet and were subjected to thermic shock treatment (90/0 °C for 1 min each and 3 repeats). The lysate was then resuspended in 200 μL of 1x Tris-EDTA pH = 8 (tris(hydroxymethyl)aminomethane-ethylenediaminetetraacetic acid) buffer (St. Louis, MO, USA); 20 μL of SDS (sodium dodecyl sulfate) 10% (Fluka, Buchs, Switzerland) was added to be then incubated (1 h, 55 °C) and vortexed. Then, 100 μL of NaCl 5 M and 80 μL of CTAB 1% were added to the suspension and incubated for 30 min at 65 °C and vortexed. The cell suspension was further treated by phenol-chloroform-isoamylic alcohol extraction and isopropanol precipitation. DNA was resuspended with Tris-EDTA buffer 0.1x and genomic DNA was analyzed in 1%-agarose electrophoresis to determine its quality, and the concentration was determined by the 260/280 nm ratio (Take3 accessory of Epoch Microplate Spectrophotometer, Bio Tek, Winooski, VT, USA).

#### 2.11. 16S rDNA Amplification and Molecular Identification

Genomic DNA was subjected to 16S rDNA amplification by PCR using 27f (5´-AGAGTTTGATCCTGGCTCAG-3´) and 1512r (5´-ACGGCTACCTTGTTACGACTT-3´) [23]. The reaction mixture was done in a final volume of 50 μL with 29 μL of sterile Milli Q water, 7 μL of 10x Taq buffer, 4 μL of each primer (10 pmol/μL), 1 μL of dNTP Mix, 1 μL of Taq Polymerase, and 4 μL of extracted genomic DNA (100 ng/μL). PCR amplification conditions were set with an initial denaturation step (95 °C, 10 min), 25 amplification cycles of denaturation (93 °C, 1 min each), annealing (50 °C, 1 min each), elongation (72 °C, 1:30 min each), and the final extension step (72 °C, 10 min). PCR products were purified and analyzed in agarose gel electrophoresis (1.5%). PCR product sequence was done using the *Taq* FS dye terminator cycle sequencing fluorescence-based sequencing method in an automated sequencer model 3730xl (Perkin Elmer/Applied Biosystems; Psomagen, Rockville, MD, USA). The homology of sequences was compared using the basic local alignment search tool (BLAST) using highly similar sequences algorithm with default search parameters of the NCBI database, optimizing for highly similar sequences (Blast version 5, dbV5). Sequences obtained were deposited in GenBank, and Accession Numbers SRX6825780 to SRX6825791 were assigned to the isolates reported in this work.

### 2.12. Statistical Analysis

All the experiments were done in triplicate, and the results were expressed as mean ± standard deviation. Cluster analysis was done based on Eucledian distance and complete linkage using Minitab 18 software (Minitab Inc., State College, PA, USA), establishing 50% of similitude as a criterion to separate clusters. Also, Pearson correlation was determined (*p* < 0.05) to compare the results of each assay.

## 3. Results

### 3.1. Presumptive LAB Isolates from Chihuahua Cheese.

Chihuahua cheese is a traditional dairy product that is manufactured by Mennonite and non-Mennonite communities in Chihuahua, Mexico, and until recently, they were using raw milk for its preparation. We reported the characterization of their manufacturing process and described the microbiological changes presented during the manufacturing process and nine-month ripening period [13,14]. Chihuahua cheese is a semi-matured cheese that includes a cheddaring step after curd formation, and salt is added after cheddaring and before pressing. Traditional production is done using raw milk, without the addition of starter cultures, but pasteurization and addition of a starter culture have been incorporated lately in most cheese factories. The presumptive LAB isolates reported here were isolated from traditional manufacturers, which used raw milk as a starting material [13,14]. From the microbial count plates, pure cultures were maintained at −20 °C (cryopreserved in 40% glycerol solution), and from this collection, the isolates from three cheese factories were analyzed. Table 1 describes the number of isolates analyzed from each dairy, including the manufacturing step or the ripening period. The identification codes of the strains included the number of the isolate, followed by an identification of the dairy farm samples. For dairy A, the letter **T** was used; for dairy B, the abbreviation **sa** was used; and for dairy E, the abbreviation **vl** was used. Dairy B is a traditional Mennonite factory, dairy A is also from Mennonite producers, but semi-industrialized and dairy E is of non-Mennonite farmers. A total of 214 isolates were analyzed from three dairy farms.

### 3.2. Enzymatic Activities of Presumptive LAB Isolates

The hydrolytic capacities of 214 presumptive LAB isolates were semi-quantitatively tested using a high-protein media to determine proteolytic capacities, and a triglyceride-supplemented media to determine lipolytic capacity. A total of 201 isolates hydrolyzed the casein-peptone medium supplemented with skimmed milk, as observed by the clear halo around the microbial growth.

**Figure 1:**
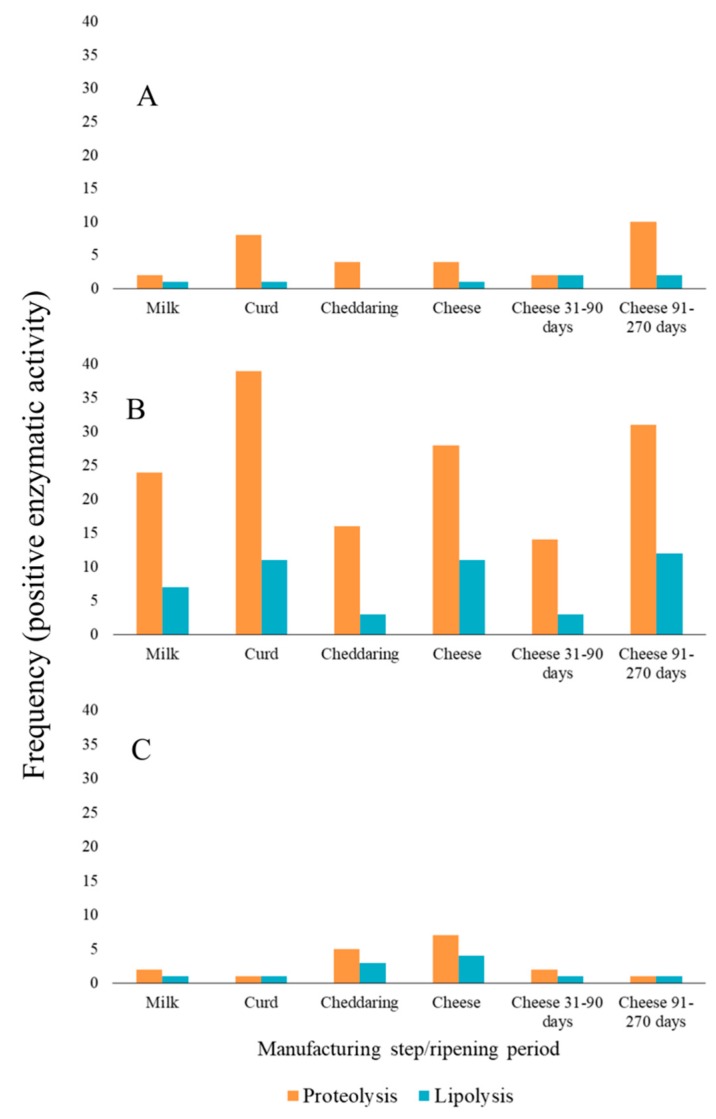
Frequencies of the qualitative enzymatic determinations of presumptive lactic acid bacteria (LAB) isolates. (**A**) dairy A; (**B**) dairy B; (**C**) dairy E from Sanchez-Gamboa et al. [13].

On the other hand, 65 isolated exhibited tributyrin hydrolysis based on the clear zone around the microbial colony. Frequencies of both enzymatic activities are observed in Figure 1, where proteolysis was consistently higher than lipolysis from the isolates of each dairy and part of the process. Contrary to proteolytic activity, lipolysis was observed after 48 h of incubation.

### 3.3. Antimicrobial Activity of Presumptive LAB Isolates

To identify antimicrobial capacity by spot-on-the-lawn (SOTL), the inhibition observed between MRS and TSAYE agar was compared. MRS media contains ten times more dextrose than TSAYE and it is expected that the production of organic acids can cause inhibition in MRS, but only the strains that presented a zone of inhibition in both media were interpreted as positive [15].

Presumptive LAB isolates tested for enzymatic activities were also tested for antimicrobial capacity against *L. monocytogenes* and *E. coli* using SOTL. No inhibition against *E. coli* was observed by any presumptive LAB isolate tested, but 85 inhibited *L. monocytogenes* by SOTL assay. From the 85 anti-listeria isolates, 39 showed an inhibition zone larger than the control strain (11.15 ± 0.3 mm). The 39 antimicrobial isolates also presented proteolytic activity, and only 16 were also lipolytic. The 39 presumptive LAB isolates were selected for confirmatory tests that included antimicrobial and antioxidant capacities of their cell-free supernatants; both properties can be associated with proteinic metabolites. The confirmatory methods used were agar diffusion assay (ADA) and the percentage of growth inhibition was determined by microplate (MP). In the ADA test, only *L. monocytogenes* presented a clear inhibition zone by 20 isolates, but all isolates showed growth inhibition with both indicator strains. Growth inhibition was in the range of 30% to 100% against *L. monocytogenes* and from 0% to 50% against *E. coli*. Only the isolates that inhibited >80% (bold case) bacterial growth in the microplate inhibition test showed clear zones in the ADA (Table 2).

The techniques used to measure antimicrobial capacity against *L. monocytogenes* presented a positive correlation between the SOTL technique and ADA (r = 0.607, *p* ≤ 0.001), as well as compared with with the % Inh (r = 0.418, *p* = 0.007); a positive correlation was also observed between ADA and % Inh measurements against *L. monocytogenes* (r = 0.666, *p* ≤ 0.001) and *E. coli* (r = 0.355, *p* = 0.025). The % Inh test also presented a correlation between the two indicator strains (r = 0.346, *p* = 0.029). No isolates were inhibitory of *E. coli* in SOTL or ADA (data not shown).

### 3.4. Radical Scavenging Capacity of the Casein Hydrolysates 

From the 214 strains that were tested for casein proteolysis, more than 90% showed proteolytic activity. The high percentage proteolytic isolates do not allow a selection screening based on this activity; therefore, the 39 potentially anti-listeria isolates (that were also proteolytic) were considered to analyze the production of antioxidant compounds. The antioxidant metabolites can be by-products of the proteolytic activity of the presumptive LAB isolates on different proteins and can act as radical scavengers. DPPH and ABTS radicals were used to test for the antioxidant activity of CFSH. As the methods were done in 60 min kinetics with readings every 10 min, the maximum peak of inhibition (40 min) was considered for the analysis (Table 2). Inhibition of DPPH radical was in the range of 6.3% to 33.2%, while ABTS radical values were from 35.5% to 68.7% of inhibition. There was a positive correlation between the DPPH and ABTS methods tested (r = 0.314, *p* = 0.048).

### 3.5. Selection of Presumptive LAB Isolates with Antimicrobial and Antioxidant Properties

**Figure 2:**
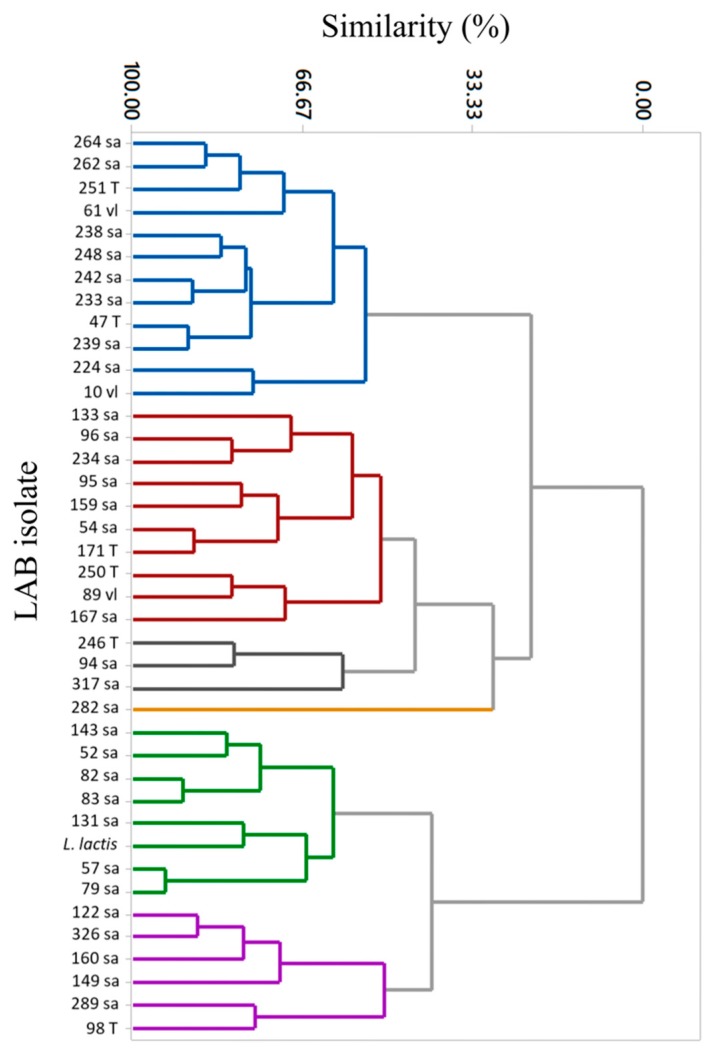
Cluster analysis of the antimicrobial and antioxidant activities from the selected presumptive LAB isolates.

In order to determine similarities among the isolates and select the best candidates for the production of antioxidant and antimicrobial metabolites, the results presented in Table 2 were used for cluster analysis (Figure 2). Six clusters were identified with 50% similarity. Regarding the identification code for the presumptive LAB isolates, the number is related to the number of isolates, and the letters are related to the dairy farm where the Chihuahua cheese was manufactured (sa refers to dairy B, T to dairy A, and vl to dairy E).

Cluster 1 (blue) grouped 12 isolates showing *L. monocytogenes* growth inhibition from 16 to 26 mm clear halos and the neutralized cell-free supernatant presented values of 784–2811 AU against *L. monocytogenes* in ADA, as well as 76%–100% inhibition in the microplate growth inhibition test; additionally, *E. coli* was inhibited in the range of 41%–47% *E. coli* strain by % Inh. Cluster one isolates also showed competitive inhibition of both radicals (11%–19% of DPPH and 50%–60% of ABTS). Seven of the 12 isolates that were grouped in cluster one were also positive for lipolysis (isolates: 10 vl, 131 sa, 167 sa, 238 sa, 239 sa, 250 T, 251 T, 262 sa, 289 sa, 47 T, 57 sa, and 61 vl).

For clusters 2–6, different inhibition patterns were observed for the antimicrobial and antioxidant tests. Cluster 2 (red) grouped 10 isolates that showed inhibition percentage of *L. monocytogenes* and *E. coli* (% Inh), but not the strains showing inhibition of *L. monocytogenes* using ADA. Three isolates (95sa, 250T and 167sa) from cluster 2 were lipolytic. Cluster 3 (gray) grouped three isolates with three isolates that showed competitive inhibition of *L. monocytogenes*, but non-competitive inhibition of *E. coli*. The same competitive anti-listeria isolates were lipolytic (246T and 94sa). Cluster 4 (yellow) includes only one strain that showed antimicrobial capabilities against both indicator microorganisms as in cluster 1, notwithstanding the inhibition of ABTS radical was the lowest percentage observed for all 39 strains and almost half from measurements of cluster 1; strain 282sa was not lipolytic. Cluster 5 (green) includes seven isolates and the control strain that were negative for antimicrobial confirmation by ADA; microplate inhibition (% Inh) of *L. monocytogenes* was almost half compared with cluster 1, as was also the inhibition of *E. coli*. Three isolates (83sa, 131sa, and 57sa) from cluster 5 showed lipolytic activity. Cluster 6 (purple) grouped six isolates that showed no inhibition of *L. monocytogenes* by ADA assay, as well as no % Inh of *E. coli* by the microplate inhibition test. This group also showed the lowest % Inh of *L. monocytogenes*. Only one (289sa) showed lipolytic activity. Clusters 6 and 5 showed the lowest levels of microbial inhibition, but the highest level of radicals inhibition.

### 3.6. Molecular Identification of Selected Presumptive LAB Isolates from Chihuahua Cheese

Table 3 shows the results for the molecular identification of the presumptive LAB isolates from Cluster 1. BLASTn analysis identified the sequences of the amplified rDNA 16S from the 12 isolates as *Enterococcus faecium.* Isolates were obtained from the Chihuahua cheese manufacturing from three dairies (sa refers to dairy B, T to dairy A, and vl to dairy E). 264sa and 262sa where milk isolated; 238sa and 242 sa were isolates obtained from the cheddaring sample; and the rest were cheese isolated from 31 to 90 days of ripening time with an exception of 251T, which was isolated from 120 days of maturation.

## 4. Discussion

Manufacturing of artisanal Chihuahua cheese includes the use of unpasteurized milk, where autochthonous LAB contributes to the transformation of milk components thanks to enzymatic processes [24]. This transformation includes the generation of a particular flavor and texture. Naturally found LAB in artisanal products are worth studying as their multiple metabolic capabilities and antimicrobials production have been already demonstrated to improve food quality.

The purpose of this study was to select presumptive LAB isolated from the Chihuahua cheese manufacturing and ripening process, with multi-functional potential, including enzymatic activity, as well as antimicrobial and antioxidant potentials. The selection was based on the antimicrobial capacity of the isolates against *L. monocytogenes* and *E. coli*, which have been linked to numerous foodborne outbreaks [25,26]. Spot-on-the-lawn was the first test used for selection; this is a widely used assay to detect the production of antimicrobial compounds. However, the culture media can influence the detection of inhibition halos by the antimicrobial action of organic acids. In the present study, the comparison between MRS and TSAYE clear zones was considered in order to determine if the inhibition halos were the result of organic acids (MRS) or other metabolites (TSAYE). Moraes et al. [27] also assay anaerobic conditions in a modified MRS media and restrict the production of organic acids and hydrogen peroxide that can also produce antagonistic activity. Some reports do not use SOTL assays as the first test for screening antimicrobial capacity; instead, they use agar diffusion test with NCFSs [28,29,30]; however, SOTL was the easiest way to test numerous isolates. The positive correlation between SOTL and ADA confirms the feasibility of using them as a first test and a confirmation assay, respectively. Our selecting criteria was based on the capacity of the presumptive LAB isolates to produce a zone of inhibition larger than the control strain used; a commercial strain that is reported to produce Nisin [31]. Our goal was to find not only new potential bacteriocin-producing strains, but also ones that could have a more significant potential than cells already reported.

Two kinds of cell free supernatant were obtained to determine the different metabolic capacities, for antimicrobial and antioxidant activities. MRS media was used to confirm the antimicrobial activity as is the most used media for bacteriocin production and identification [32,33], because its composition meets LAB exigent nutritional requirements. On the other hand, casein-milk supplemented media was used to study the production of antioxidant components because of the presence of casein and milk proteins, which are the most studied protein compounds for bioactive peptides’ production [34].

Growth inhibition assays were done as a confirmatory assay using NCFSs, considering that bacteriocins are peptides secreted during microbial growth [35]. The results of antimicrobial measurement could be negatively affected by agar-based tests, owing to the diffusion of hydrophobic and amphipathic compounds [36]; some bacteriocins are considered highly hydrophobic peptides as nisin, but there are also bacteriocins with amphiphilic structures, which vary their amounts of hydrophobicity [37]. Differences observed between the antimicrobial assays could be attributed to the nature of the tested compounds for the agar-based tests; this was confirmed by the lack of inhibition halo from the nisin-producer control strain by ADA; however, the control strain can inhibit both indicator strains by the microplate technique (% Inh), which is not agar-based. This is also confirmed by the positive correlation among SOTL and ADA, the agar-based methods. The percentage of growth inhibition in the MP assay allowed the confirmation of the antimicrobial activity against both indicator strains; it was also observed that, only when growth inhibition was higher than 70%, the ADA test was also positive. This could be related to the mechanism of action of the antimicrobial compounds; for example, if they are bactericidal or bacteriostatic compounds; however, this could be demonstrated using serial dilutions and kinetic inhibition studies [38].

The identification of proteolytic and lipolytic activity was evaluated in an agar-based technique using a high protein supplemented media and a natural triglyceride added for each case, in order to test presumptive LAB isolates qualitatively. It was expected to have a large number of proteolytic isolates, in agreement with the naturally protein-rich environment from where they were isolated. Similar studies found that a high percentage of dairy isolated LAB strains are proteolytic [39,40]. Compared with the proteolytic activities, fewer isolates showed lipolytic activity. Even though only a qualitative study was developed in the present report, this provides a future perspective of 65 isolates in which the characterization of lipase or/and esterase activity could be developed, as there are only a few reports of lipolytic LAB isolates [41].

The proteolytic action of LAB strains can be used to obtain bioactive peptides that could participate as regulators in physiological processes [4,5,6]. Although the antimicrobial, proteolytic, and lipolytic properties of LAB have been extensively reported, the selection of strains that have more than one capacity can lead to the detection of multifunctional LAB strains, as we reviewed previously [42]. Antioxidant activities were demonstrated by the inhibition of the ABTS^+^ and DPPH^+^ radicals, using the supernatants obtained by presumptive LAB fermentation of casein-peptone broth. Casein-skimmed milk media has already been used to study the production of antioxidant peptides; however, each strain or even the breaking down method (e.g., enzymatic) could have different patterns of hydrolysis and release different peptides with different bioactivities [43]. The molecular weight, the amino acid composition and sequence, as well as the secondary structure and stability of the bioactive peptides obtained by proteolytic activity of the isolates are important factors that affect antioxidant properties [44]; thus, further studies are needed to explain differences in antioxidant activity. Comparison of inhibition percentages between already published reports is complicated because the methods used and the concentration of the radicals used varied. In one report, bovine casein hydrolysates obtained by tryptic treatment showed 13% of DPPH^+^ inhibition [34] compared with the results presented in this study (6%–33%). However, the authors used the radical in a higher concentration (six times higher) and used a method that where no implementation of changes (as in the present work) to avoid protein precipitation, which could affect the interpretation of the results.

*Enterococcus* isolates are commonly isolated from dairy products, especially from artisanal processes; there are, therefore, many reports regarding their functional properties, which have been associated to metabolic capacities that can play an important role in the technological improvement of food processing. Simultaneous production of enzymes and antimicrobial compounds from *Enterococcus* strains have been already reported [10], but the results presented in this report are focused on the identification of multi-functional isolates of LAB naturally present in the artisanal Chihuahua cheese manufacturing process. The selected isolates can be used for the production of metabolites that can be added to food products for better quality. LAB strains with proteolytic and antimicrobial activities can be used to generate a multi-functional cell-free product, by a one-strain/single process, avoiding the negative impact of adding *Enterococcus* live strains to food products. Further characterization is required to elucidate the nature of the compounds responsible for the antimicrobial and antioxidant activity, as well as their mechanism of action.

## 5. Conclusions

Screening of autochthonous presumptive LAB isolates obtained from Chihuahua cheese by specific agar-based methods for enzymatic capacity and antimicrobial capacity by the spot-on-the-lawn method allowed the selection of 39 isolates. On the basis of the antimicrobial and antioxidant capacity of their cell-free supernatants, 12 isolates were further selected and identified by molecular-based methodology as *Enterococcus faecium*. Presumptive LAB strains present more than one capacity, including antimicrobial and proteolysis or lipolysis, and are, therefore, candidates for further studies. Multi-functional LAB strains can be used to generate products derived from their fermentation activity, which can be used to improve the quality and safety of food products.

## Figures and Tables

**Table 1:** Presumptive lactic acid bacteria (LAB) isolates from the Chihuahua cheese manufacturing and ripening process, with identification of the number of isolates from each condition.

Manufacturing/ Ripening	Dairy		
	A	B	E
Milk	2	26	2
Curd	10	40	1
Cheddaring	5	16	6
Cheese	4	29	7
Cheese 30–90 days	2	14	2
Cheese 91–270 days	11	36	1
Total	34	161	19

Dairy identification A = T; B = sa; E = vl. Dairy identification is the same as in [13,14].

**Table 2:** Antimicrobial and antioxidant activity of 39 selected presumptive LAB isolates from Chihuahua cheese. DPPH, 1,1-diphenyl-2-picrylhydrazyl; ABTS, 2,2-azinobis (3-ethylbenzothiazoline-6-sulfonate).

	Antimicrobial Test				Antioxidant Test	
Bacterial Isolate	*L. monocytogenes*			*E. coli*	DPPH (%)	ABTS (%)
	mm	AU	% Inh	% Inh		
**264 sa**	19.85 ± 0.41	1445 ± 278	**92.11 ± 1.24**	47.90 ± 0.41	11.07 ± 1.21	59.68 ± 1.66
**262 sa**	20.16 ± 0.41	784 ± 26	**93.73 ± 1.38**	44.13 ± 4.46	15.71 ± 1.96	60.74 ± 8.51
251 T	23.37 ± 0.25	1010 ± 129	76.45 ± 6.23	41.37 ± 4.31	13.95 ± 1.93	62.07 ± 2.30
**61 vl**	20.16 ± 0.53	1692 ± 127	**81.74 ± 1.59**	45.26 ± 1.52	23.86 ± 1.32	64.46 ± 1.22
**238 sa**	16.81 ± 0.27	2625 ± 192	**91.66 ± 1.69**	48.78 ± 0.41	11.32 ± 2.20	66.05 ± 0.46
**248 sa**	19.76 ± 0.5	2811 ± 211	**88.60 ± 2.25**	46.53 ± 0.66	17.09 ± 0.95	66.05 ± 0.92
**242sa**	17.58 ± 0.31	2611 ± 174	**87.48 ± 2.5**	47.95 ± 3.37	15.46 ± 4.02	61.01 ± 2.11
**233 sa**	16.40 ± 0.75	2258 ± 179	**85.90 ± 0.96**	49.26 ± 1.27	19.97 ± 6.04	61.01 ± 2.11
**47 T**	20.29 ± 0.77	2365 ± 86	**90.70 ± 2.24**	41.22 ± 6.29	12.95 ± 2.85	58.62 ± 1.38
**239 sa**	18.55 ± 0.41	2152 ± 362	**101.15 ± 0.72**	42.28 ± 5.92	12.45 ± 2.14	59.68 ± 1.66
**224 sa**	26.26 ± 0.43	1866 ± 58	**94.91 ± 0.25**	44.09 ± 4.71	12.83 ± 1.89	56.76 ± 2.79
**10 vl**	22.27 ± 0.58	2294 ± 0.58	**90.86 ± 2.49**	46.80 ± 1.54	14.83 ± 2.39	50.13 ± 3.59
133 sa	18.25 ± 0.47	0 ± 0	56.96 ± 5.27	46.71 ± 5.84	12.95 ± 3.24	64.19 ± 4.43
96 sa	15.57 ± 0.73	0 ± 0	49.56 ± 7.12	49.03 ± 4.62	17.34 ± 1.89	51.99 ± 8.17
234 sa	18.8 ± 0.1	0 ± 0	66.37 ± 3.44	42.29 ± 1.32	14.46 ± 2.17	53.85 ± 2.39
95 sa	11.62 ± 0.28	0 ± 0	69.72 ± 1.42	39.40 ± 5.92	20.48 ± 2.07	57.82 ± 4.43
159 sa	16.09 ± 0.85	0 ± 0	65.04 ± 8.85	28.46 ± 17.4	21.86 ± 6.90	54.91 ± 2.00
**54 sa**	15.48 ± 0.1	577 ± 161	**80.77 ± 0.14**	38.49 ± 8.78	24.24 ± 1.74	57.82 ± 11.28
**171 T**	14.40 ± 0.42	767 ± 15.33	**96.01 ± 1.64**	42.08 ± 4.92	24.74 ± 2.10	58.36 ± 6.03
**250 T**	18.46 ± 0.6	797 ± 77	**96.87 ± 0.85**	36.51 ± 11.2	6.30 ± 1.00	61.54 ± 5.97
**89 vl**	16.34 ± 0.3	105 ± 7	**92.08 ± 2.76**	43.8 ± 2.64	8.69 ± 4.41	64.99 ± 0.80
**167 sa**	12.56 ± 0.67	121 ± 38	**98.22 ± 1.87**	45.15 ± 3.59	11.70 ± 4.41	58.36 ± 3.59
**246 T**	15.67 ± 0.74	1094 ± 49	**92.96 ± 2.58**	−7.11 ± 8.49	12.70 ± 3.76	55.44 ± 3.65
**94 sa**	12.99 ± 0.22	89 ± 46	**101.68 ± 2.05**	−5.41 ± 5.8	13.20 ± 1.21	56.76 ± 2.56
317 sa	11.23 ± 0.37	0 ± 0	53.94 ± 16.76	13.74 ± 9.43	13.08 ± 1.99	49.34 ± 1.22
282 sa	17.07 ± 0.79	1141 ± 343	**93.38 ± 0.93**	41.08 ± 7.62	14.58 ± 2.47	35.54 ± 1.38
143 sa	17.56 ± 0.06	0 ± 0	46.93 ± 3.09	55.74 ± 2.23	22.99 ± 1.32	66.84 ± 0.46
52 sa	15.18 ± 0.8	0 ± 0	44.50 ± 8.52	50.73 ± 2.63	29.76 ± 1.74	68.17 ± 0.00
82 sa	14.58 ± 0.82	0 ± 0	51.58 ± 3.38	50.48 ± 2.91	26.62 ± 2.10	61.27 ± 3.22
83 sa	15.30 ± 0.29	0 ± 0	48.44 ± 2.79	46.96 ± 2.4	23.74 ± 4.91	58.36 ± 7.52
131 sa	12.36 ± 0.72	0 ± 0	47.85 ± 5.29	31.1 ± 14.22	21.98 ± 5.01	67.11 ± 1.84
*L. lactis*	11.15 ± 0.3	0 ± 0	55.74 ± 5.42	46.43 ± 1.55	18.09 ± 4.23	68.44 ± 0.92
57 sa	11.50 ± 0.22	0 ± 0	58.82 ± 3.8	46.86 ± 8.45	31.89 ± 7.41	64.99 ± 1.59
79 sa	11.17 ± 0.33	0 ± 0	60.17 ± 3.82	39.62 ± 2.78	33.15 ± 2.30	66.05 ± 0.46
122 sa	14.08 ± 0.58	0 ± 0	37.98 ± 5.24	2.70 ± 9.93	10.19 ± 2.14	67.11 ± 1.22
326 sa	14.55 ± 0.37	0 ± 0	50.24 ± 5.92	2.34 ± 2.56	12.20 ± 3.06	63.13 ± 1.84
160 sa	16.11 ± 0.21	0 ± 0	32.71 ± 17	−11.28 ± 6	20.23 ± 2.10	60.21 ± 3.18
149 sa	17.62 ± 0.23	0 ± 0	53.11 ± 0.62	−7.25 ± 6.09	14.08 ± 2.56	59.15 ± 6.67
289 sa	12.81 ± 0.18	0 ± 0	55.28 ± 4.41	−2.12 ± 4.49	27.25 ± 5.88	66.31 ± 2.00
98 T	16.19 ± 0.36	0 ± 0	36.29 ± 0.9	−10 ± 7.6	33.27 ± 5.78	68.70 ± 1.22

SOTL, spot-on-the-lawn; ADA, agar diffusion assay; AU, arbitrary units; % Inh, percentage of inhibition.

**Table 3:** Molecular identification of 12 selected LAB isolates.

Strain	Blast Identification	Accession Number
264sa	*Enterococcus faecium*	**SRX6825780**
238sa	*Enterococcus faecium*	**SRX6825781**
248sa	*Enterococcus faecium*	**SRX6825784**
224sa	*Enterococcus faecium*	**SRX6825785**
242sa	*Enterococcus faecium*	**SRX6825786**
262sa	*Enterococcus faecium*	**SRX6825787**
233sa	*Enterococcus faecium*	**SRX6825788**
47T	*Enterococcus faecium*	**SRX6825789**
61vl	*Enterococcus faecium*	**SRX6825790**
10vl	*Enterococcus faecium*	**SRX6825791**
251T	*Enterococcus faecium*	**SRX6825782**
239sa	*Enterococcus faecium*	**SRX6825783**

## References

[1] Law B.A., Kolstad J. (1983). Proteolytic systems in lactic acid bacteria. Antonie Van Leeuwenhoek.

[2] Meisel H., Bockelmann W. (1999). Bioactive peptides encrypted in milk proteins: proteolytic activation and thropho-functional properties. Antonie Van Leeuwenhoek.

[3] Aluko R., Aluko R. (2012). Bioactive peptides. Functional Foods and Nutraceuticals.

[4] Osuntoki A., Korie I. (2010). Antioxidant activity of whey from milk fermented with *Lactobacillus* species isolated from Nigerian fermented foods. Food Technol. Biotechnol..

[5] Stanisavljević N.S., Vukotić G.N., Pastor F.T., Sužnjević D., Jovanović Ž.S., Strahinić I.D., Fira D.A., Radović S.S. (2015). Antioxidant activity of pea protein hydrolysates produced by batch fermentation with lactic acid bacteria. Arch. Biol. Sci..

[6] Rizzello C.G., Lorusso A., Russo V., Pinto D., Marzani B., Gobbetti M. (2017). Improving the antioxidant properties of quinoa flour through fermentation with selected autochthonous lactic acid bacteria. Int. J. Food Microbiol..

[7] Tsakalidou E., Manolopoulou E., Kabaraki E., Zoidou E., Pot B., Kersters K., Kalantzopoulos G. (1994). The combined use of whole-cell protein extracts for the identification (SDS-PAGE) and enzyme activity screening of lactic acid bacteria isolated from traditional Greek dairy products. Syst. Appl. Microbiol..

[8] Mierau I., Kleerebezem M. (2005). 10 years of the nisin-controlled gene expression system (NICE) in *Lactococcus lactis*. Appl. Microbiol. Biotechnol..

[9] Smaoui S., Elleuch L., Bejar W., Karray-Rebai I., Ayadi I., Jaouadi B., Mathieu F., Chouayekh H., Mellouli L. (2010). Inhibition of fungi and gram-negative bacteria by bacteriocin BacTN635 produced by *Lactobacillus plantarum* sp. TN635. Appl. Biochem. Biotechnol..

[10] Arthur T.D., Cavera V.L., Chikindas M.L. (2014). On bacteriocin delivery systems and potential applications. Future Microbiol..

[11] Ramakrishnan V., Balakrishnan B., Rai A.K., Narayan B., Halami P.M. (2012). Concomitant production of lipase, protease and enterocin by *Enterococcus faecium* NCIM5363 and *Enterococcus durans* NCIM5427 isolated from fish processing waste. Int. Aquat. Res..

[12] Sharma D., Singh Saharan B. (2014). Simultaneous production of biosurfactants and bacteriocins by probiotic *Lactobacillus casei* MRTL3. Int. J. Microbiol..

[13] Sánchez-Gamboa C, Hicks-Pérez L., Gutiérrez-Méndez N., Heredia N., García S., Nevárez-Moorillón G. (2018). Microbiological changes during ripening of Chihuahua cheese manufactured with raw milk and its seasonal variations. Foods.

[14] Sánchez-Gamboa C., Hicks-Pérez L., Gutiérrez-Méndez N., Heredia N., García S., Nevárez-Moorillón G.V. (2018). Seasonal influence on the microbial profile of Chihuahua cheese manufactured from raw milk. Int. J. Dairy Technol..

[15] Lewus C.B., Montville T.J. (1991). Detection of bacteriocins produced by lactic acid bacteria. J. Microbiol. Methods.

[16] Avaiyarasi N.D., Ravindran A.D., Venkatesh P., Arul V. (2016). In vitro selection, characterization and cytotoxic effect of bacteriocin of *Lactobacillus sakei* GM3 isolated from goat milk. Food Control.

[17] Carrazco-Palafox J., Rivera-Chavira B.E., Ramírez-Baca N., Manzanares-Papayanopoulos L.I., Nevárez-Moorillón G.V. (2018). Improved method for qualitative screening of lipolytic bacterial strains. MethodsX.

[18] Pownall T.L., Udenigwe C.C., Aluko R.E. (2010). Amino acid composition and antioxidant properties of pea seed (*Pisum sativum* L.) enzymatic protein hydrolysate fractions. J. Agric. Food Chem..

[19] Nicklisch S.C., Waite J.H. (2014). Optimized DPPH assay in a detergent-based buffer system for measuring antioxidant activity of proteins. MethodsX.

[20] Re R., Pellegrini N., Proteggente A., Pannala A., Yang M., Rice-Evans C. (1999). Antioxidant activity applying an improved ABTS radical cation decolorization assay. Free Radic. Biol. Med..

[21] Delgado-García M., Contreras-Ramos S.M., Rodríguez J.A., Mateos-Díaz J.C., Aguilar C.N., Camacho-Ruíz R.M. (2018). Isolation of halophilic bacteria associated with saline and alkaline-sodic soils by culture dependent approach. Heliyon.

[22] Delgado-García M., Flores-Gallegos A.C., Kirchmayr M., Rodríguez J.A., Mateos-Díaz J.C., Aguilar C.N., Muller M., Camacho-Ruíz R.M. (2019). Bioprospection of proteases from *Halobacillus andaensis* for bioactive peptide production from fish muscle protein. Electron. J. Biotechnol..

[23] Merzoug M., Dalache F., Karam H.Z., Karam N.E. (2016). Isolation and preliminary characterisation of bacteriocin produced by *Enterococcus faecium* GHB21 isolated from Algerian paste of dates “ghars”. Ann. Microbiol..

[24] Nájera-Domínguez C., Gutiérrez-Méndez N., Nevárez-Moorillon G., Caro-Canales I. (2014). Comparison of volatile compounds produced by wild *Lactococcus lactis* in miniature Chihuahua-type cheeses. Dairy Sci. Technol..

[25] CDC Outbreak of *Listeria* Infections. https://www.cdc.gov/listeria/outbreaks/monocytogenes-08-19/index.html.

[26] CDC Reports of *E. coli* Outbreak Investigations from 2019. https://www.cdc.gov/ecoli/2019-outbreaks.html.

[27] Moraes P.M., Perin L.M., Ortolani M.B.T., Yamazi A.K., Viçosa G.N., Nero L.A. (2010). Protocols for the isolation and detection of lactic acid bacteria with bacteriocinogenic potential. LWT-Food Sci. Technol..

[28] Yang E., Fan L., Jiang Y., Doucette C., Fillmore S. (2012). Antimicrobial activity of bacteriocin-producing lactic acid bacteria isolated from cheeses and yogurts. AMB Express.

[29] Grosu-Tudor S.S., Stancu M.M., Pelinescu D., Zamfir M. (2014). Characterization of some bacteriocins produced by lactic acid bacteria isolated from fermented foods. World J. Microbiol. Biotechnol..

[30] Hwanhlem N., Chobert J.M., Aran H. (2014). Bacteriocin-producing lactic acid bacteria isolated from mangrove forests in southern Thailand as potential bio-control agents in food: Isolation, screening and optimization. Food Control.

[31] Punyauppa-path S., Phumkhachorn P., Rattanachaikunsopon P. (2015). Nisin: production and mechanism of antimicrobial action. Int. J. Curr. Res. Rev..

[32] Pal A., Ramana K.V., Bawa A.S. (2010). Simplification and optimization of deMan Rogosa Sharpe (MRS) medium for enhanced production of bacteriocin by *Weissella paramesenteroides* DFR-8. J. Food Sci. Technol..

[33] Gautam N., Sharma N., Ahlawat O.P. (2014). Purification and characterization of bacteriocin produced by *Lactobacillus brevis* UN isolated from Dhulliachar: a traditional food product of north east India. Indian J. Microbiol..

[34] Irshad I., Kanekanian A., Peters A., Masud T. (2015). Antioxidant activity of bioactive peptides derived from bovine casein hydrolysate fractions. J. Food Sci. Technol..

[35] Kaur R., Tiwari S.K. (2018). Membrane-acting bacteriocin purified from a soil isolate *Pediococcus pentosaceus* LB44 shows broad host-range. Biochem. Biophys. Res. Commun..

[36] Bonev B., Hooper J., Parisot J. (2008). Principles of assessing bacterial susceptibility to antibiotics using the agar diffusion method. J. Antimicrob. Chemother..

[37] Rea M.C., Ross R.P., Cotter P.D., Hill C. (2011). Classification of bacteriocins from Gram-positive bacteria. Prokaryotic Antimicrobial Peptides.

[38] Vijayakumar P., Muriana P. (2015). A microplate growth inhibition assay for screening bacteriocins against *Listeria monocytogenes* to differentiate their mode-of-action. Biomolecules.

[39] El-Ghaish S., Dalgalarrondo M., Choiset Y., Sitohy M., Ivanova I., Haertlé T., Chobert J.M. (2010). Screening of strains of *lactococci* isolated from Egyptian dairy products for their proteolytic activity. Food Chem..

[40] Liu M., Bayjanov J.R., Renckens B., Nauta A., Siezen R.J. (2010). The proteolytic system of lactic acid bacteria revisited: a genomic comparison. BMC Genom..

[41] Akabanda F., Owusu-Kwarteng J., Tano-Debrah K., Parkouda C., Jespersen L. (2014). The use of lactic acid bacteria starter culture in the production of Nunu, a spontaneously fermented milk product in Ghana. Int. J. Food Sci..

[42] Venegas-Ortega M.G., Flores-Gallegos A.C., Martínez-Hernández J.L., Aguilar C.N., Nevárez-Moorillón G.V. (2019). Production of Bioactive Peptides from Lactic Acid Bacteria: A Sustainable Approach for Healthier Foods. Compr. Rev. Food Sci. Food Saf..

[43] Kliche T., Li B., Bockelmann W., Habermann D., Klempt M., de Vrese M., Wutkowski A., Clawin-Raedecker I., Heller K.J. (2017). Screening for proteolytically active lactic acid bacteria and bioactivity of peptide hydrolysates obtained with selected strains. Appl. Microbiol. Biotechnol..

[44] Zou T.B., He T.P., Li H.B., Tang H.W., Xia E.Q. (2016). The structure-activity relationship of the antioxidant peptides from natural proteins. Molecules.

